# Identifying hub genes and immune infiltration of osteoarthritis using comprehensive bioinformatics analysis

**DOI:** 10.1186/s13018-021-02796-6

**Published:** 2021-10-20

**Authors:** Zheng-yuan Wu, Gang Du, Yi-cai Lin

**Affiliations:** 1 Department of Hand Plastic Surgery, The First People’s Hospital of Linping District, No. 369, Linping Yingbin Road, Yuhang District, Hangzhou, 311199 Zhejiang China; 2 Department of Bone and Joint Surgery, The First Affiliated Hospital of Guangxi Medical University, No. 22 Shuangyong Road, Nanning, 530021 Guangxi China

**Keywords:** Osteoarthritis, Gene expression omnibus, Immune infiltration, Bioinformatics analysis

## Abstract

**Background:**

Osteoarthritis (OA) is the most common chronic degenerative joint disorder globally that is characterized by synovitis, cartilage degeneration, joint space stenosis, and sub-cartilage bone hyperplasia. However, the pathophysiologic mechanisms of OA have not been thoroughly investigated.

**Methods:**

In this study, we conducted various bioinformatics analyses to identify hub biomarkers and immune infiltration in OA. The gene expression profiles of synovial tissues from 29 healthy controls and 36 OA samples were obtained from the gene expression omnibus database to identify differentially expressed genes (DEGs). The CIBERSORT algorithm was used to explore the association between immune infiltration and arthritis.

**Results:**

Eighteen hub DEGs were identified as critical biomarkers for OA. Through gene ontology and pathway enrichment analyses, it was found that these DEGs were primarily involved in PI3K-Akt signaling pathway and Rap1 signaling pathway. Furthermore, immune infiltration analysis revealed differences in immune infiltration between patients with OA and healthy controls. The hub gene ZNF160 was closely related to immune cells, especially mast cell activation in OA.

**Conclusion:**

Overall, this study presented a novel method to identify hub DEGs and their correlation with immune infiltration, which may provide novel insights into the diagnosis and treatment of patients with OA.

## Introduction

Osteoarthritis (OA) is a common arthritis disease worldwide that can severely impair the function of joints and affect the quality of life of the older population [1, 2]. OA is a progressive disorder that is characterized by the degradation of hyaline articular cartilage, along with subchondral sclerosis, narrowing of the joint space, osteophyte formation, synovial hyperplasia, and structural alterations of peripheral muscles and ligaments [3]. The main clinical manifestations of OA are knee dysfunction and local pain. Approximately, 10% of the world’s population aged over 60 years has symptomatic OA [4]. Patients with OA always pose considerable psychological, financial, and physical burdens.

Thus, because of such serious implications, many scientists are focusing their attention on investigating the pathogenesis and mechanisms underlying OA. According to its etiology, OA can be divided into two categories: primary and secondary forms [5]. In the primary form, aging plays a major role in OA occurrence and progression. Meanwhile, certain diseases, such as chondromalacia patellae, erosive OA, and primary generalized OA, are also regarded as subsets of idiopathic OA. In the secondary form, any predisposing factors that can breach the integrity of the cartilage matrix have the underlying potential to induce OA, such as joint trauma, obesity, significant family history, reduced levels of sex hormones, and muscle weakness [6]. Among these, joint trauma and obesity are considered as the strongest risk factors [7]. OA development in the knee joint tissues is significantly connected with physical activity, lifestyle, and weight, whereas in case of hip OA, it is mainly related to age, weight, sex, genetic factors, trauma, and occupation [8]. The development mechanism of OA correlation with mechanical, biochemical, and cellular processes [6]. As cartilage matrix proteolysis begins, the chondrocytes become prone to erosion and fibrillation, and collagen fragments and proteoglycans are released into the synovial fluid. This process results in the inflammatory response of the synovium tissues, and further promotes cartilage thin out, joint space narrowing, and spurs outgrowth. In recent years, studies have provided a deeper understanding of the pathophysiology of OA. However, there are only a few screening biomarkers and therapeutic interventions that are of significance for the clinical treatment of OA. Therefore, the elucidation of more unique OA biomarkers is urgently needed for accurately identifying patients and developing therapies.

With the development of microarray technologies, bioinformatics analyses have been widely employed to identify disease-specific biomarkers, explore significant epigenetic and genetic alterations, and reveal the molecular mechanism of arthritis. Integrated bioinformatical analyses of multiple expression cohorts have revealed several hub genes, including AKT1, IL2, TP53, CD247, and CCL5, that commonly participate in the development of both OA and rheumatoid arthritis [9, 10]; these genes may act as therapeutic targets for arthritis therapy. In addition, the interaction relationship between these genes has also been clearly revealed by previous studies. Several biological pathways (such as osteoclast differentiation, inflammation, and immune response) are also commonly associated with arthritis progression [10]. Furthermore, the TNF signaling pathway, instead of the chemokine signaling pathway, is considered as the main pathway involved in OA inflammatory development [11]. These findings, thus, provide novel insight into the exploration of arthritis. However, only few bioinformatics studies have solely focused on OA and its correlation with immune cells. The weighted gene co-expression network (WGCN), which focuses on gene sets rather than individual gene expression, is a frequently used method to understand the gene association patterns between different phenotypic traits [12, 13]. During WGCN analysis (WGCNA), OA expression data can be used to construct a powerful scale-free network to identify hub biomarkers for mechanism evaluation and clinical diagnosis. Furthermore, differential gene expression analysis of transcriptional data is another powerful tool that provides quantitative expression level changes between two subgroups [14]. The combination of these two bioinformatic analyses has been previously used for tumor gene identification, such as in case of osteosarcoma [15], gastric cancer [16], and head and neck squamous cell carcinoma [17], but rarely for arthritis diseases.

In the present study, we identified differentially expressed genes (DEGs) between healthy controls and patients with OA using two approaches, namely, WGCNA and differential gene expression analysis, to enhance the discriminatory ability of highly connected genes. Subsequently, the co-expressed differentially expressed hub genes from the two groups were used to identify the same pathological manifestations or mechanisms in OA. Furthermore, CIBERSORT algorithm method was used to analyze the content of 22 immune cells in synovium tissues and explore the connections between the expression of hub biomarkers and immune infiltration in OA. Thus, the aims of this study were to identify co-expressed hub genes, characterize immune infiltration differences in the synovium of OA, and provide novel insights into the diagnosis and therapeutic targets of OA.

## Materials and methods

### Raw data acquisition and preprocessing

Gene expression profiles of 136 synovial tissues from joints were acquired from the gene expression omnibus (GEO) database from the following cohorts: GSE12021, GSE55235, GSE55457, and GSE55584 (https://www.ncbi.nlm.nih.gov/geo/). After separating rheumatoid arthritis tissues and samples whose detection platform was different from that of others, as in GSE12021 (GPL96, Affymetrix Human Genome U133B), the remaining diseased specimens (including 29 samples from healthy controls and 36 samples from patients with OA) were all tested using the same Affymetrix Human Genome U133A platform and merged as one profile to explore the hub genes. All pathological synovial tissues were collected from patients with OA after joint replacement surgery, and normal synovium was collected early postmortem from macroscopically normal knee joints. Approval by an ethics committee was not necessary because all data were collected from publicly available databases. The merged gene expression profiles were log2-transformed and then normalized using the “sva” R package to remove batch effects, as described in previous studies [11, 18].

### Construction of the WGCN and hub module identification

The “WGCNA” package in R was utilized to construct a gene co-expression network [19, 20]. WGCNA is an advanced systems biology-based approach to identifying functionally enriched gene groups and providing more consistent gene rankings. Further, its focus on module eigengenes effectively circumvents the multiple testing problems of standard differential expression methods. Pairwise Pearson’s correlation coefficients were calculated for the genes, and a weighted adjacency matrix was created using the following formula: amn =|cmn|β (cmn = Pearson’s correlation between gene m and gene n; amn = adjacency between genes m and n). Following this, a suitable soft-threshold parameter “β” was selected to emphasize strong gene correlations and penalize weak correlations. The adjacencies were then transformed to a topological overlap matrix (TOM). Using TOM-based dissimilarity measures, the average linkage hierarchical clustering was used to construct the gene dendrogram with a minimum module size of 50; the dissimilarity of the module eigengenes was also calculated. Furthermore, two parameters, module eigengenes and gene significance, were identified to reveal the modules that were relevant to clinical traits of OA; consequently, the genes within the functional module were considered to be candidate genes.

### Differential expression analysis and interaction with the modules of interest

Differential expression analysis was performed using the “limma” R package to identify candidate DEGs, using the significance analysis of microarrays with a false discovery rate (FDR) < 0.05 and |log2 fold change (FC)|> 1 [21]; the results were visualized as a volcano plot using the “ggplot2” R package [22]. Furthermore, the overlapping genes between candidate DEGs and OA-WGCNA were extracted and considered as the “real” DEGs; these were visualized in a Venn diagram using the “VennDiagram” R package [23].

### Gene ontology (GO) and Kyoto Encyclopedia of genes and genomes (KEGG) enrichment analyses

KEGG pathway and GO enrichment analyses were applied to investigate the biological functions of identified DEGs using DAVID version 6.8 [24]. Three terms comprised the GO analysis, including cellular component (CC), biological process (BP), and molecular function (MF). Both the FDR and *p* values < 0.05 were considered as significant terms.

### Immune infiltration analysis by CIBERSORT

The CIBERSORT algorithm (https://cibersort.stanford.edu/) is a widely applied method for calculating the proportions of 22 types of leukocytes in complex tissues profiled by microarray [25]. In this study, all samples were screened with a *p* value < 0.05, and CIBERSORT analysis was performed to characterize immune cell infiltration in OA synovial tissues. The composition of immune cells in each sample was visualized using a bar plot and heatmap, and the infiltration levels of each cell between patients with OA and healthy controls were analyzed using the “vioplot” R package.

## Results

### DEG identification

**Fig. 1:**
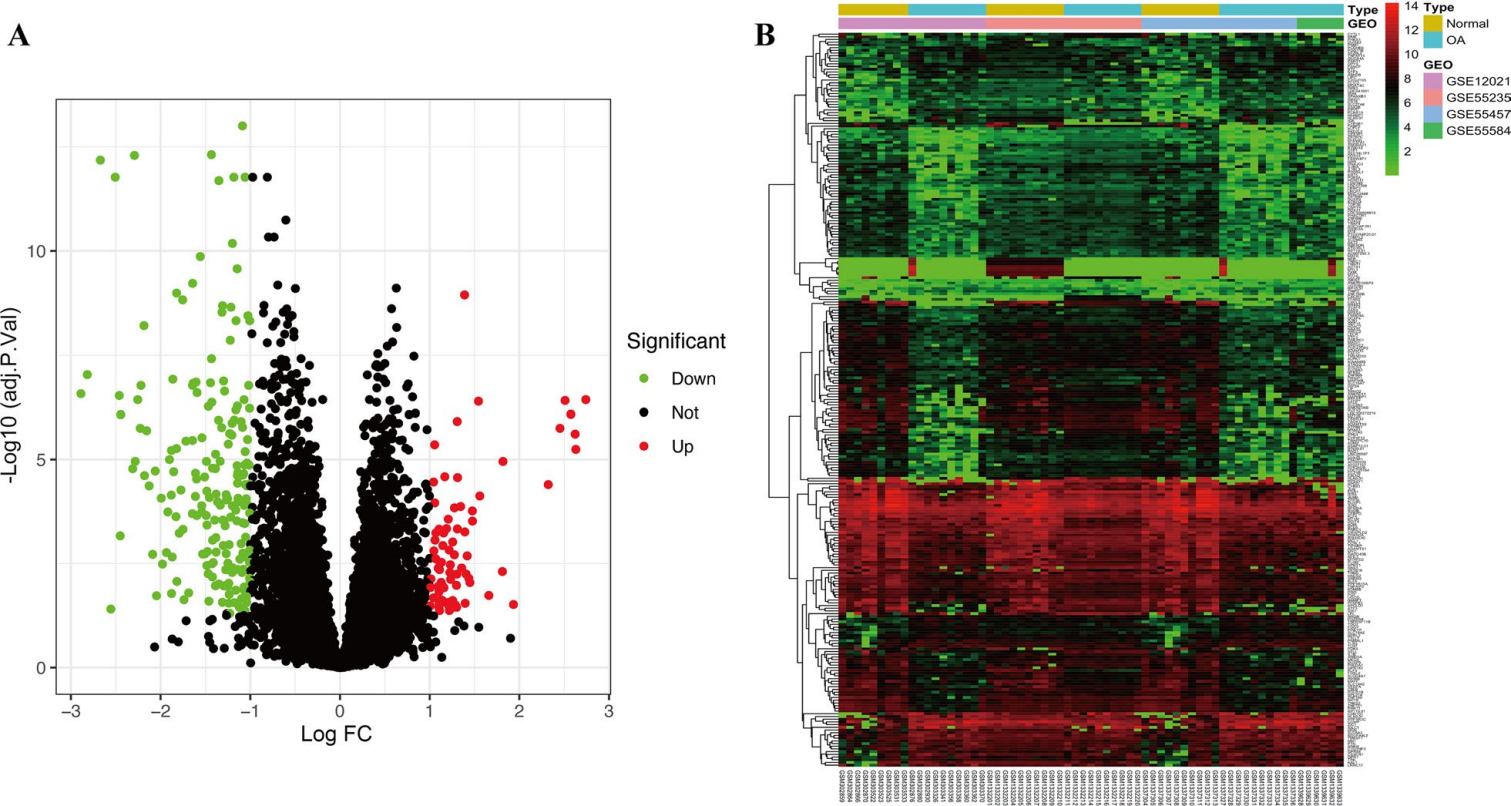
Identification of differently expressed OS genes. Volcano plot (**A**) and heatmap (**B**) of DEGs between OA synovial and normal controls

After processing the raw microarray results from GSE12021, GSE55457, GSE55235, and GSE55584, candidate DEGs were screened using the criteria of FDR < 0.05 and |log2 FC|> 1. As a result, a total of 271 DEGs, comprising 194 downregulated and 77 upregulated genes, were differentially expressed between the OA samples and controls (Fig. 1A, B).

### Functional enrichment analysis of the candidate DEGs

GO analysis showed that in terms of BP, the candidate DEGs were significantly enriched in response to multi-multicellular organism processes, regulation of chemokine production, female pregnancy, cellular response to external stimulus, and chemokine production (Table 1). With respect to MF, the candidate DEGs were mainly enriched in receptor ligand activity, DNA-binding transcription activator activity, cytokine activity, major histocompatibility class II receptor activity, and growth factor activity. With respect to pathway enrichment, KEGG pathway analysis showed that the candidate DEGs were mainly enriched in the TNF signaling pathway, rheumatoid arthritis, osteoclast differentiation, interleukin-17 signaling pathway, and mitogen-activated protein kinase signaling pathway (Table 2).

**Table 1:** GO enrichment analysis of DEGs (top 10 terms of each category were listed)

Ontology	ID	Description	Adj. *p* value	Count
BP	GO:0044706	Multi-multicellular organism process	0.00087884	14
BP	GO:0032642	Regulation of chemokine production	0.00087884	9
BP	GO:0007565	Female pregnancy	0.00087884	13
BP	GO:0071496	Cellular response to external stimulus	0.00093422	17
BP	GO:0032602	Chemokine production	0.00101597	9
BP	GO:0050727	Regulation of inflammatory response	0.00139524	20
BP	GO:0009314	Response to radiation	0.00139524	19
BP	GO:0031667	Response to nutrient levels	0.00139524	20
BP	GO:0050900	Leukocyte migration	0.00139524	20
BP	GO:0045444	Fat cell differentiation	0.00139524	13
MF	GO:0048018	Receptor ligand activity	0.00159324	20
MF	GO:0001228	DNA-binding transcription activator activity, RNA polymerase II-specific	0.01065086	17
MF	GO:0005125	Cytokine activity	0.01837692	11
MF	GO:0032395	MHC class II receptor activity	0.02077473	3
MF	GO:0008083	Growth factor activity	0.02077473	9
MF	GO:0005126	Cytokine receptor binding	0.02077473	12
MF	GO:0008201	Heparin binding	0.02077473	9
MF	GO:0071889	14–3-3 protein binding	0.02776368	4
MF	GO:0017017	MAP kinase tyrosine/serine/threonine phosphatase activity	0.02788606	3
MF	GO:0005539	Gycosaminoglycan binding	0.03279933	10

**Table 2:** Top 10 pathways of DEGs enriched in KEGG analysis

ID	Description	Adj. *p* value	Count
hsa04668	TNF signaling pathway	1.48E−06	13
hsa05323	Rheumatoid arthritis	0.00081662	9
hsa04380	Osteoclast differentiation	0.00113721	10
hsa04657	IL-17 signaling pathway	0.00342316	8
hsa04010	MAPK signaling pathway	0.00351106	14
hsa04933	AGE-RAGE signaling pathway in diabetic complications	0.00351106	8
hsa05140	Leishmaniasis	0.00351106	7
hsa04064	NF-kappa B signaling pathway	0.00351106	8
hsa05169	Epstein-Barr virus infection	0.00396226	11
hsa05164	Influenza A	0.00401821	10

### Identification of hub modules by constructing a WGCN

**Fig. 2:**
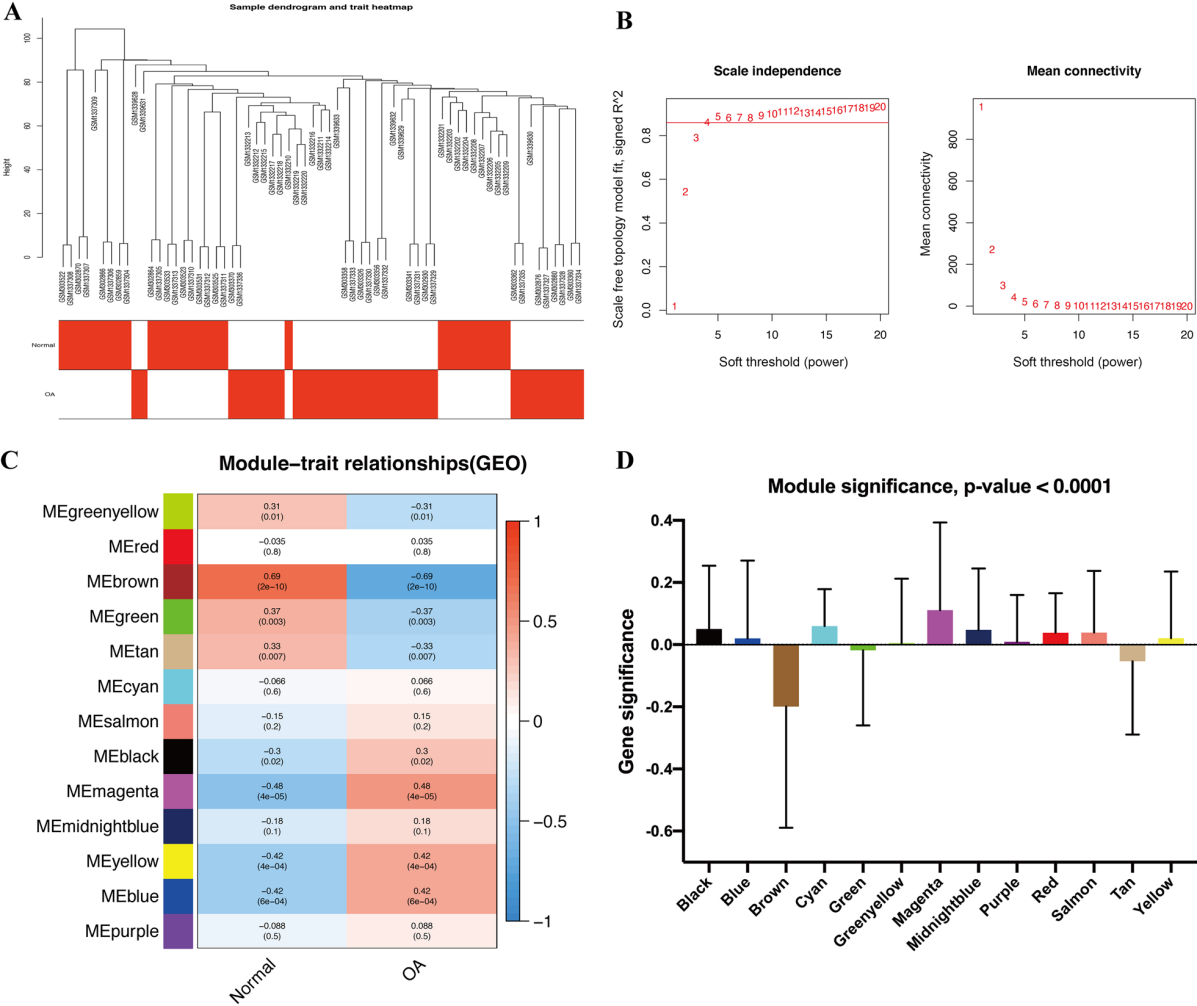
Identification of modules associated with the clinical information in the OA tissues. **A** Clustering dendrogram of OA samples and normal controls. **B** The scale-free fit index for soft-thresholding powers. **C** A heatmap showing the correlation between the gene module and clinical trait (OA and normal). Each module was assigned with different colors. The correlation coefficient in each cell represented the correlation between gene module and the clinical traits, which decreased in size from red to blue. **D** Distribution of average gene significance and errors in the modules associated with OA progression

**Fig. 3:**
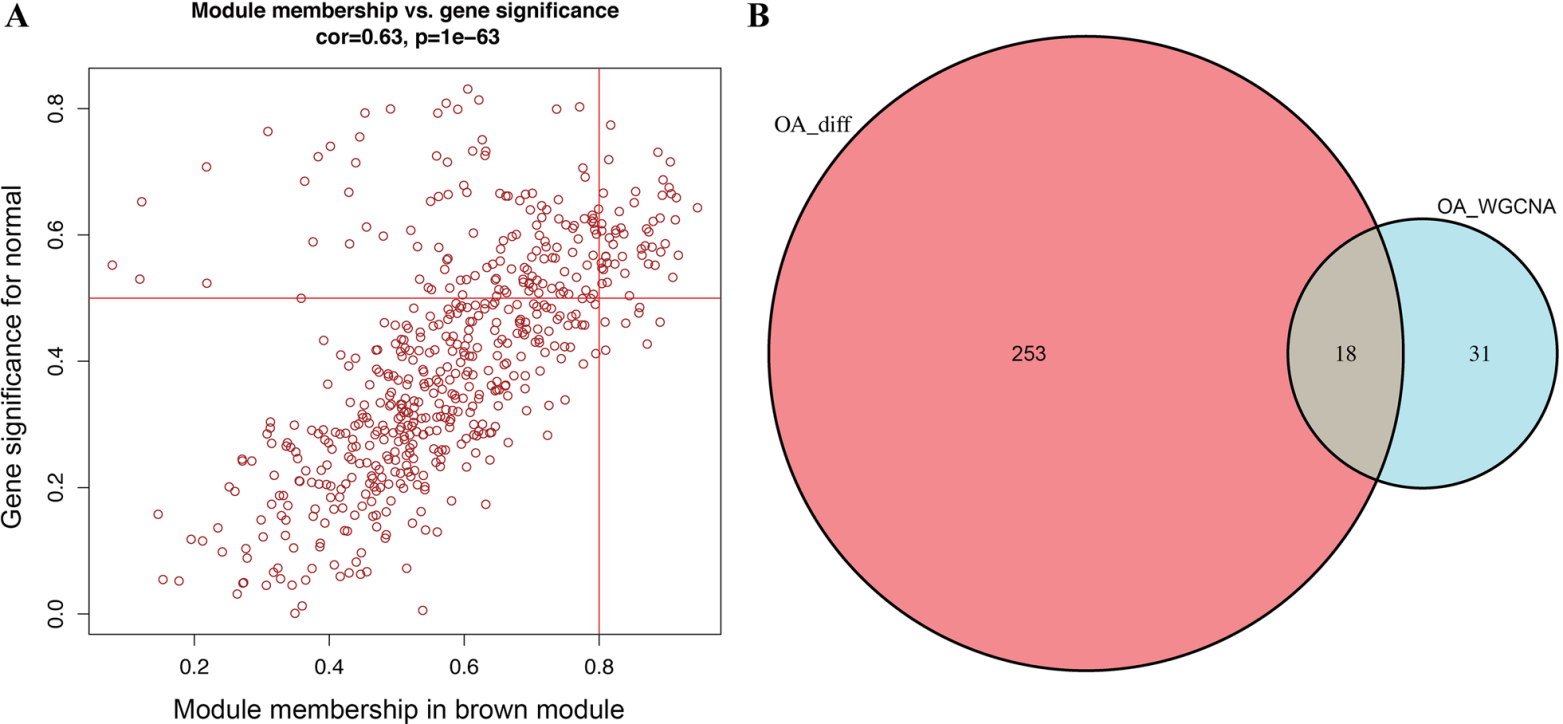
Identification of real DEGs among the OA synovial tissues. **A** Scatter plot of module eigengenes in brown modules. **B** The Venn diagram of genes among candidate DEGs and WGCNA of interest genes from brown module

**Fig. 4:**
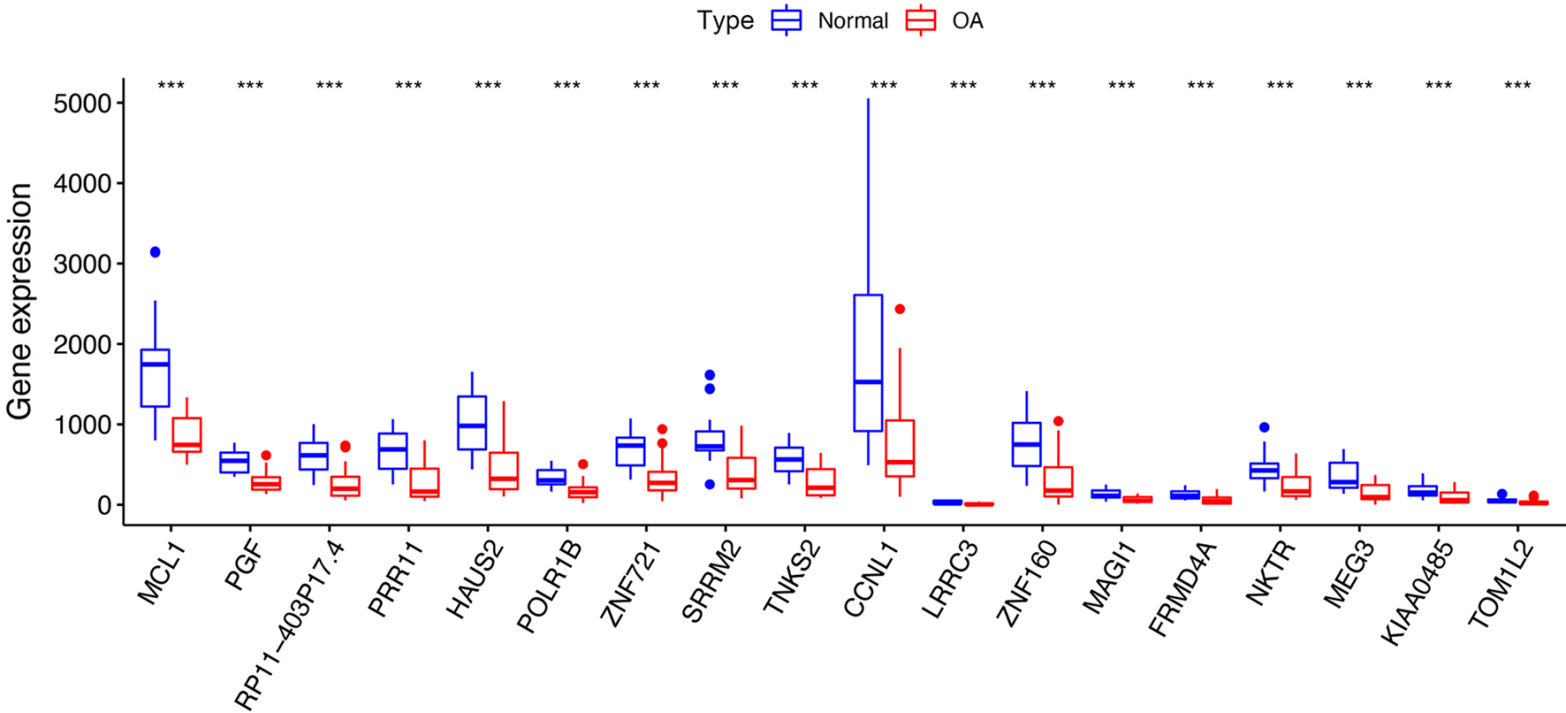
Box plot shows mRNA expression pattern of identified hub DEGs in normal tissues and OA synovial samples

To identify the functional clusters in OA, WGCNA was performed for 36 OA tissues. As shown in Fig. 2A, synovial tissues from healthy joints and OA joints were included in the analysis. To construct a scale-free network, *β* = 4 (scale-free *R*^2^ = 0.86) was selected as the soft threshold (Fig. 2B), and a total of 13 co‑expressed modules were identified (Fig. 2C). Subsequently, each module was assigned a different color to identify the connections between the module and two clinical traits (normal and OA). Among all the modules, the brown module was found to have the closest association with OA development (Fig. 2D). Among this module, 49 genes with remarkable connectivity (MM > 0.8 and GS > 0.5) were identified as significant hub genes of interest (Fig. 3A). The number distribution of the co-expressed genes from the DEGs and hub genes from the brown module of OA is shown in Fig. 3B. A total of 18 overlapping genes were extracted for further prognosis analysis. The gene expression level comparison of the hub genes indicated that all 18 genes were significantly downregulated in the OA samples (Fig. 4).

### GO and KEGG enrichment analyses of hub biomarkers

**Fig. 5:**
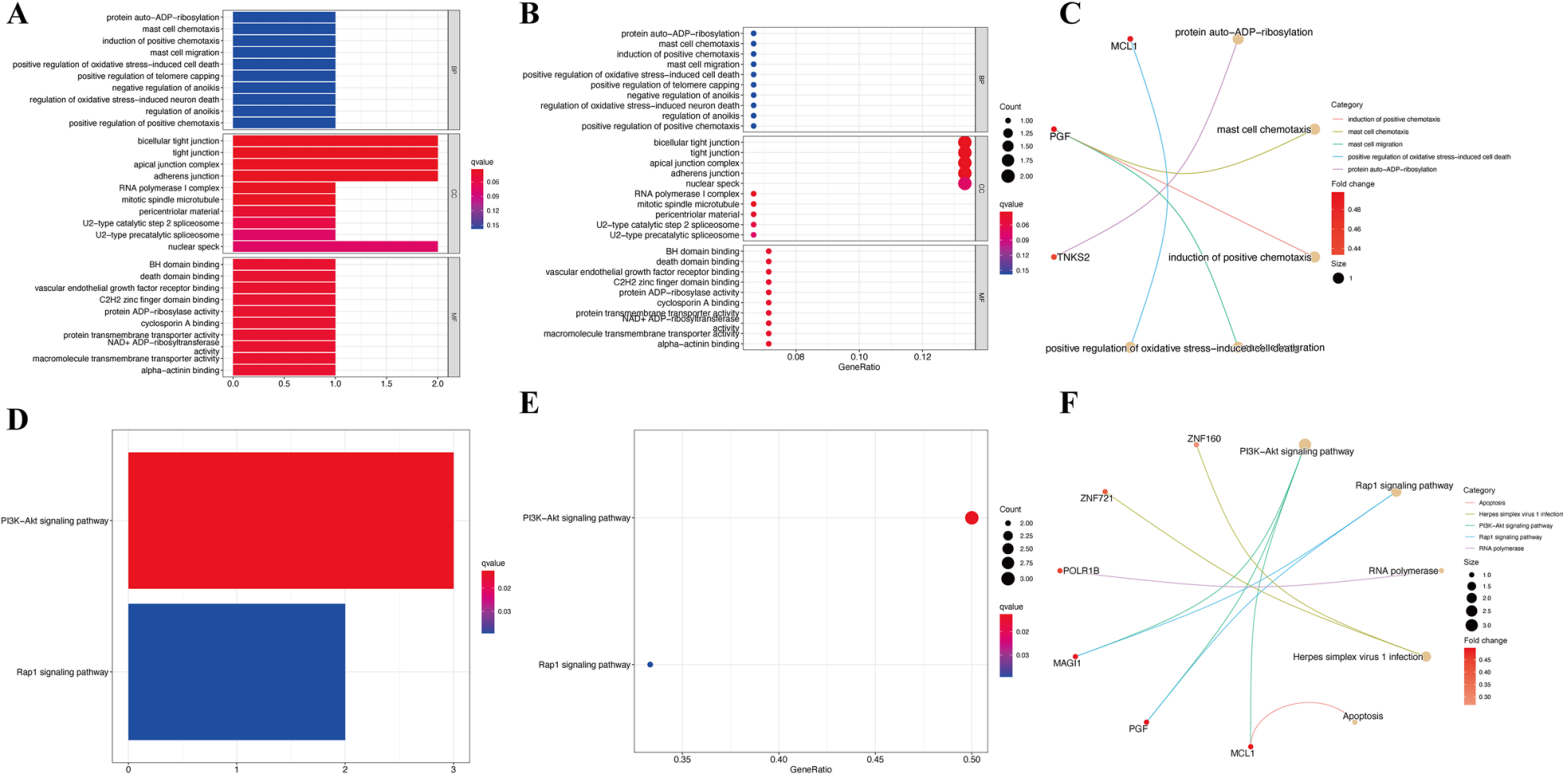
Functional enrichment analysis of identified hub DEGs. (**A**–**C**) GO enrichment terms of hub genes in biological process (BP), cellular component (CC), and molecular function (MF). (**D**–**F**) KEGG enrichment terms of hub genes. In each bubble plot, the size of the dot represents the number of enriched genes

As shown in Fig. 5A–C, the 18 identified hub biomarkers were primarily involved in some critical BPs, such as protein auto-ADP-ribosylation, mast cell chemotaxis and migration, and induction of positive chemotaxis. The hub genes were also found to be enriched in tight junction, apical junction complex, and adherens junction in the CC category and BH domain binding, death domain binding, and vascular endothelial growth factor receptor binding in the MF category. Moreover, the results of KEGG enrichment analysis indicated that the selected hub genes played a critical role in numerous pathways, such as PI3K-Akt signaling pathway and Rap1 signaling pathway (Fig. 5D–F).

### Correlation of *ZNF160* with immune infiltration in OA

**Fig. 6:**
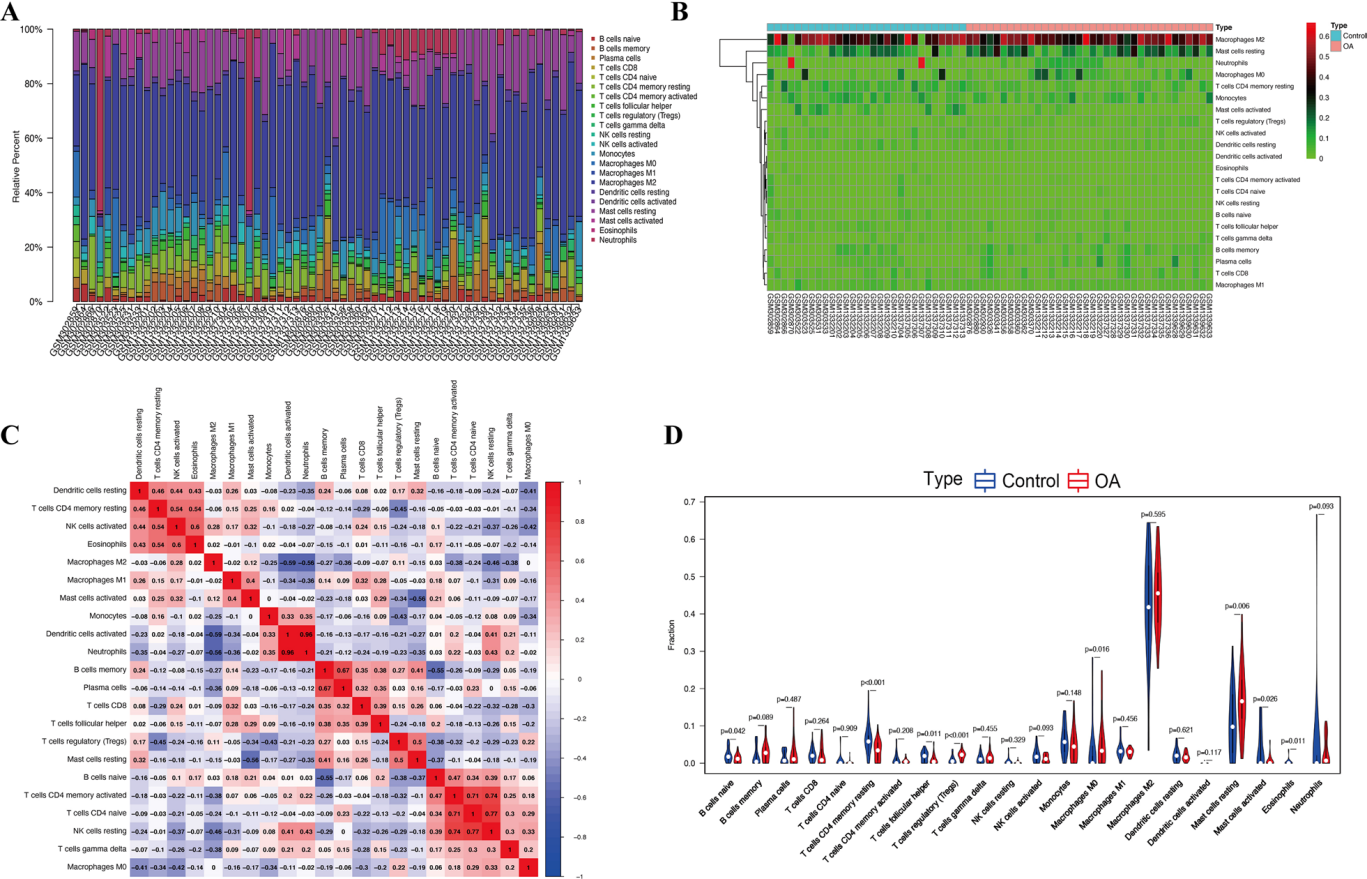
Immune infiltration analysis performed in OA tissues. Barplot (**A**) and heatmap (**B**) showed the composition of 22 subpopulations of immune cells in 36 OA synovial and 29 normal controls. **C** Heatmap showed the correlation between 22 kinds of immune cells in OA and numeric in each tiny box indicating the *p* value of correlation between two types of cells. **D** The violin plot showed the difference of immune infiltration between OA (red color) and normal controls (blue color)

**Fig. 7:**
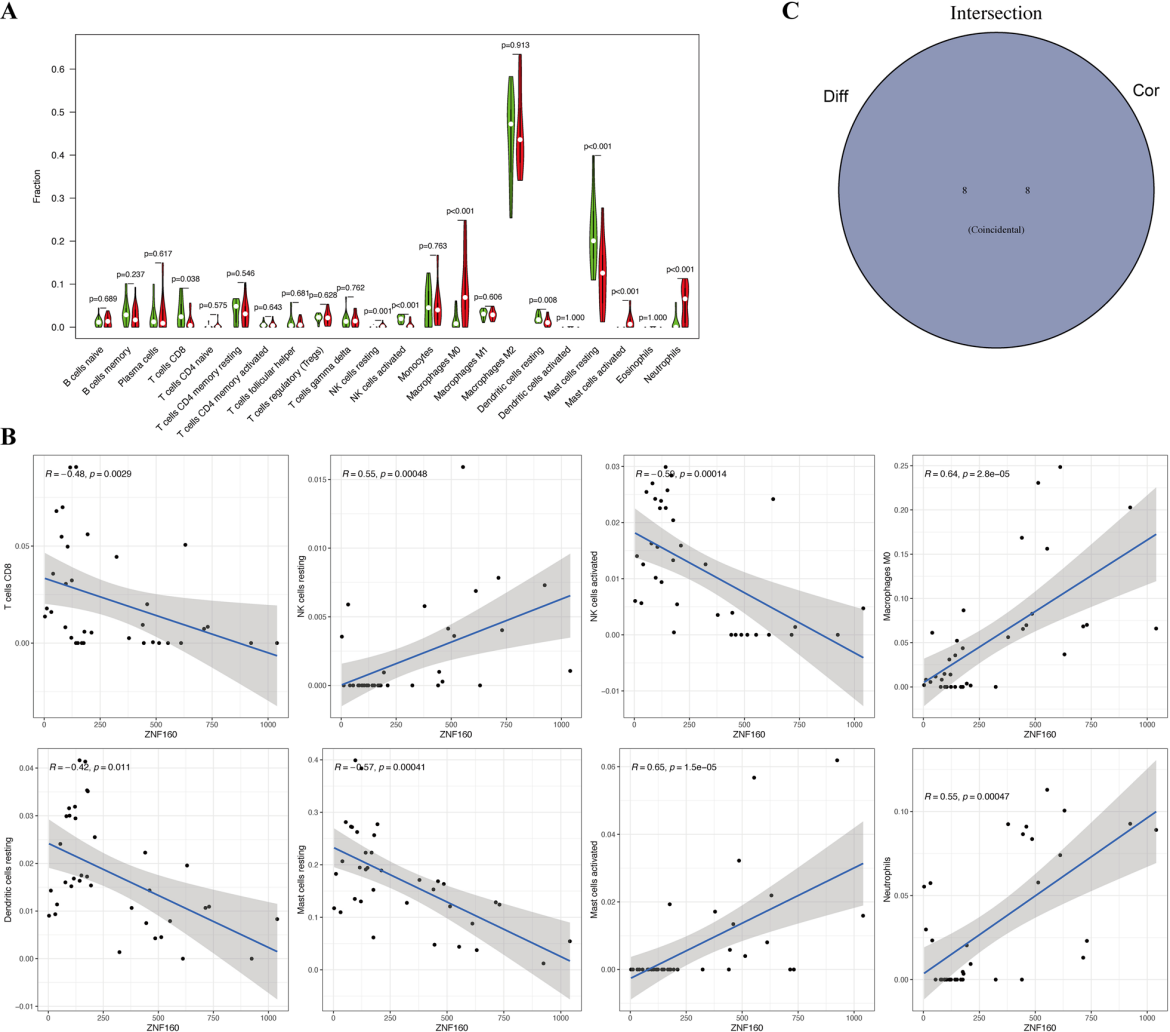
Correlation of immune infiltration with *ZNF160* expression in OA. **A** Violin plot showed the ratio differentiation of 22 kinds of immune cells between OA samples with low (green color) or high (red color) *ZNF160* expression. **B** Scatter plot showed the correlation of eight kinds of immune cells proportion with the *ZNF160* expression (*p* < 0.05). The blue line in each plot was fitted linear model indicating the proportion tropism of the immune cell along with *ZNF160* expression. **C** Venn plot of immune cells codetermined by the difference and correlation analysis

The zinc finger protein 160 (*ZNF160*) gene showed the highest fold change among the hub genes (|log2 FC|= − 1.9) and was thus selected for further exploration. To further confirm the interactions between *ZNF160* expression and immune infiltration, the CIBERSORT algorithm was used to analyze the proportion of 22 immune cell types in OA. As shown in Fig. 6, the OA samples tended to have a lower proportion of naïve B cells, CD4 memory resting T cells, follicular helper T cells, and eosinophils, and a higher proportion of regulatory T cell (Tregs), M0 macrophages, resting mast cells, and activated mast cells. Meanwhile, the difference and correlation analysis of OA samples also revealed that the infiltrating levels of eight types of immune cells were significantly associated with *ZNF160* expression levels (*p* < 0.05; Fig. 7). Among them, activated mast cells, resting NK cells, macrophages, and neutrophils were positively correlated with *ZNF160* expression, whereas activated NK cells, CD8 T cells, resting dendritic cells, and resting mast cells were negatively associated with *ZNF160* expression. These results indicated that *ZNF160* might participate in the progression of OA through modulation of immune cell infiltration levels.

## Discussion

OA, the most common osteoarthropathy disease, results in irreversible bone erosion and cartilage destruction. Without timely and effective treatment, OA can severely affect the function of a patient’s joints [26]. In recent years, increasing studies have focused on the modulation effects of synovitis in OA. Synovial inflammation leads to the formation of inflammatory pannus, resulting in cartilage erosion and a negative impact on the therapies [27, 28]. Evaluating the potential mechanism of synovitis in OA may help improve individual treatment and disorder diagnosis. The development of high-throughput technologies has enabled scientists to discover novel therapeutic targets and acquire a deeper understanding of molecular mechanisms of several diseases [29], including OA; however, the distinctive pathogenesis and detailed mechanism of OA progression in the synovium remains elusive.

To fill the current knowledge gap, gene profiles of 65 synovial tissues were collected, co-expression networks were constructed, and differential expression analysis was performed to identify DEGs. As a result, 18 genes, including *ZNF160*, leucine rich repeat containing 3 (*LRRC3*), proline rich 11 (*PRR11*), maternally expressed 3 (*MEG3*), cyclin L1 (*CCNL1*), pre-mRNA processing factor 31-403P17.4 (*RP11-403P17.4*), HAUS augmin like complex subunit 2 (*HAUS2*), FERM domain containing 4A (*FRMD4A*), zinc finger protein 721 (*ZNF721*), *KIAA0485*, tankyrase 2 (*TNKS2*), serine/arginine repetitive matrix 2 (*SRRM2*), target of Myb1-like 2 membrane trafficking protein (*TOM1L2*), RNA polymerase I subunit B (*POLR1B*), natural killer cell triggering receptor (*NKTR*), MCL1 apoptosis regulator (*MCL1*), membrane-associated guanylate kinase, WW and PDZ domain containing 1 (*MAGI1*), and placental growth factor (*PGF*) were identified as differentially expressed hub biomarkers. As a vital number of imprinted DLK-MEG3 locus, *MEG3* is proved to be involved not only in the modulation of immune cells [30] but also in the suppression of various bone diseases, especially OA [31]. MEG2 is significantly downregulated in the OA tissues than in the normal cartilage [32]. Meanwhile, studies found that *MEG2* exerted its inflammation inhibitory effect by inhibiting angiogenesis [32], interacting with miRNAs [33, 34], promoting the role of methylene blue [35], etc. Thus, *MEG3* has been recently regarded as an inhibitor of OA progression. *MCL1* is another biomarker that plays a vital role in the arthritis joints. As a member of the pro-survival Bcl-2 subfamily, *MCL1* is over-expressed and contributes to suppressing chronic inflammation in rheumatoid arthritis by enhancing the resistance ability of synovial fibroblasts to apoptosis [36, 37]. Although the specific role of *MCL1* in osteoarthritis is merely known, a study has discovered that *MCL1* could serve as a target of miR203 to inhibit cartilage degeneration [38]. This result indicated that our identified genes may act as hub biomarkers in the pathological progression of OA and thus warrant further exploration.

The identified DEGs were also assessed using functional enrichment analysis that revealed that most genes were closely related to PI3k-Akt and Rap1 signaling pathways [39–41], both of which are involved in the progression of arthritis. At the same time, GO analysis also indicated that these genes participated in several immune progressions, such as mast cell chemotaxis and migration. Considering the role of immune cell infiltration in OA, we further used the CIBERSORT algorithm method used to analyze OA. Similar to the previous analysis [18], several immune cells were noted to be significantly different between the OA synovium and control. Among these cells, activated mast cells, resting NK cells, macrophages, neutrophils, activated NK cells, CD8 T cells, resting dendritic cells, and resting mast cells were significantly correlated with *ZNF160* expression levels, which suggested that *ZNF160* might modulate OA synovial hyperplasia and progression by acting on these three types of immune cells. However, the exact relationship between *ZNF160* and these immune cells and the exact effect of *ZNF160* on synovial immune infiltration must be confirmed with further studies.

Nonetheless, despite these findings, there were still certain limitations in this study. Firstly, this study was performed as a retrospective analysis, thus, more prospective approaches are required to confirm the results. Secondly, there was a lack of experimental explorations performed to confirm the results we uncovered through bioinformatics. Mechanistic insight into the identified genes and their connections with related pathways should also be investigated. Finally, the physiological role of the identified hub genes in modulating immune cells is yet to be determined. In the future, experiments should be performed to achieve mechanistic insight into the identified genes and their connection with the development of OA.

## Conclusion

To summarize, a total of 18 hub genes that possibly play a critical role in OA pathogenesis were identified. The functional enrichment analysis of the identified biomarkers provided a potential mechanism for clarifying OA development. Moreover, our results showed that *ZNF160* might be an indicator for the modulation of immune cells in OA synovial tissues. Therefore, further investigation should be conducted to verify the accuracy of a combined analysis of *ZNF160* expression level and immune infiltration profiles in patients with arthritis.

## Data Availability

All gene expression profiles are available from the GEO (https://www.ncbi.nlm.nih.gov/geo) database.

## Abbreviations

OA: osteoarthritis
GEO: gene expression omnibus
DEGs: differentially expressed genes
WGCN: weighted gene co-expression network
WGCNA: WGCN analysis
TOM: topological overlap matrix
SAM: significance analysis of microarrays
FDR: false discovery rate
FC: fold change
GO: gene ontology
KEGG: Kyoto Encyclopedia of genes and genomes
DAVID: database for annotation, visualization, and integrated discovery
BP: biological process
CC: cellular component
MF: molecular function
MHC: major histocompatibility
TNF: tumor necrosis factor
IL: interleukin
*ZNF160*: zinc finger protein 160
*LRRC3*: leucine rich repeat containing 3
*PRR11*: proline rich 11
*MEG3*: maternally expressed 3
*CCNL1*: cyclin L1
*RP11-403P17.4*: pre-mRNA processing factor 31-403P17.4
*HAUS2*: HAUS augmin like complex subunit 2
*FRMD4A*: FERM domain containing 4A
*ZNF721*: zinc finger protein 721
*TNKS2*: tankyrase 2
*SRRM2*: serine/arginine repetitive matrix 2
*TOM1L2*: target of Myb1-like 2 membrane trafficking protein
*POLR1B*: RNA polymerase I subunit B
*NKTR*: natural killer cell triggering receptor
*MCL1*: MCL1 apoptosis regulator
*MAGI1*: membrane-associated guanylate kinase, WW and PDZ domain containing 1
*PGF*: placental growth factor
